# Esophageal cancer in the ICU: a clinical-epidemiological retrospective study

**DOI:** 10.1186/cc14713

**Published:** 2015-09-28

**Authors:** Fabrício RT de Carvalho, Antônio Paulo Nassar, Lucas C Macedo, Pedro Caruso

**Affiliations:** 1 Unidade de Terapia Intensiva, Ac Camargo Cancer Center, SP, Brazil

## Introduction

Esophageal cancer is a serious clinical condition with high mortality and morbidity, and during its evolution ICU admissions are common [1]. The main reasons for those admissions are elective postoperative conditions or clinical complications, such as bronchial aspiration owing to dysphagia, esophageal perforation and mediastinitis [2]. Despite the complexity of the disease, few data are available regarding esophageal cancer and critical care medicine.

## Objective

To evaluate clinical and epidemiological characteristics of such a population during their ICU stay.

## Methods

We performed a retrospective analysis of all cases with esophageal cancer that were admitted to the ICU of a large teaching hospital specializing in cancer in São Paulo, Brazil, between September 2009 and December 2014. Clinical and epidemiological characteristics of the patients were described, as well as risk factors identified regarding mortality during the ICU stay.

## Results

A total of 228 patients were analyzed during the period. Mean age was 60 ± 12 years and 82.5 % were male. Of the admissions, 50.4 % were related to elective surgery monitoring, and 49.6 % were clinical and emergency surgery admissions. The most frequent diagnosis that motivated ICU admission was postoperative monitoring (56.1 %), followed by acute respiratory failure (11.4 %) and severe sepsis (7.5 %). The mean value of SAPS 3 at admission was 59.3 ± 15.3. Overall mortality was 20.6 % during the ICU stay; 12.8 % were women and 87.2 % men. Logistic regression identified that the only independent variable related with mortality was SAPS 3 at admission (OR: 0.03-0.14; 95 % CI, *p *= 0.003).

## Conclusion

Patients with esophageal cancer admitted to the ICU are predominantly men, admitted after a surgical procedure (elective or emergency). The only risk factor identified for ICU mortality was the value of SAPS 3 at admission. Other risk factors such as age, diagnosis of admission and organ dysfunction may be related to mortality but were not observed in our patients. Further studies regarding this issue, especially prospective studies, should be performed.
